# Regional hippocampal diffusion abnormalities associated with subfield‐specific pathology in temporal lobe epilepsy

**DOI:** 10.1002/epi4.12357

**Published:** 2019-09-13

**Authors:** Sarah Treit, Graham Little, Trevor Steve, Tom Nowacki, Laura Schmitt, B. Matt Wheatley, Christian Beaulieu, Donald W. Gross

**Affiliations:** ^1^ Department of Biomedical Engineering Faculty of Medicine and Dentistry University of Alberta Edmonton Canada; ^2^ Division of Neurology Faculty of Medicine & Dentistry University of Alberta Edmonton Canada; ^3^ Department of Laboratory Medicine and Pathology Faculty of Medicine and Dentistry University of Alberta Edmonton Canada; ^4^ Department of Surgery Faculty of Medicine and Dentistry University of Alberta Edmonton Canada

**Keywords:** hippocampal sclerosis, histology, mean diffusivity, memory, temporal lobe epilepsy

## Abstract

**Objective:**

Hippocampal sclerosis (HS) is the most common pathology and best predictor of surgical outcome for medically refractory patients with temporal lobe epilepsy (TLE). Current clinical MRI methods can detect HS, but subfield pathology is poorly characterized, limiting accurate prediction of seizure‐free outcomes after surgery. Diffusion tensor imaging (DTI) can probe regional microstructural changes associated with focal hippocampal pathology, but is typically limited by low‐resolution whole‐brain acquisitions.

**Methods:**

High‐resolution (1 × 1 × 1 mm^3^) DTI, T1, and quantitative T2 of the hippocampus was acquired in 18 preoperative TLE patients and 19 healthy controls. Diffusion images were qualitatively assessed for loss of internal architecture, and whole‐hippocampus diffusion, volume, and quantitative T2 were compared across groups. Regional hippocampal diffusion abnormalities were examined in all subjects and compared to histology in four subjects who underwent anterior temporal lobectomy.

**Results:**

High‐resolution mean diffusion‐weighted images enabled visualization of internal hippocampal architecture, used to visually identify HS with 86% specificity and 93% sensitivity. Mean diffusivity (MD) elevations were regionally heterogenous within the hippocampus and varied across TLE patients. The spatial location of diffusion abnormalities corresponded with the location of focal subfield neuron loss, gliosis, and reduced myelin staining abnormalities identified with postsurgical histology in four subjects who underwent anterior temporal lobectomy. Whole‐hippocampus MD and T2 relaxation times were higher, and fractional anisotropy (FA) and volumes were lower in TLE patients relative to controls. Left hippocampus MD correlated with verbal memory in the TLE group.

**Significance:**

Visualization of internal architecture and focal diffusion abnormalities on high‐resolution diffusion imaging suggests potential clinical utility of diffusion imaging in TLE and may have significant implications for surgical planning and prediction of seizure‐free outcomes in individual patients.

**Key Points**

- Visual loss of internal architecture on high‐resolution diffusion‐weighted images is sensitive and specific to hippocampal sclerosis
- Regional diffusion abnormalities are present within the hippocampus and vary across TLE patients
- Focal areas of elevated mean diffusivity correspond with postsurgical histology in a subset of TLE patients

## 1 INTRODUCTION

Temporal lobe epilepsy (TLE) is the most common focal epilepsy syndrome and one of the most refractory to medical therapy.^1^ Anterior temporal lobectomy (ATL) has the potential to provide seizure freedom;^2^ however, long‐term outcome studies demonstrate seizure recurrence in 50% of patients,^3^ highlighting the need to develop better prognostic markers for patient selection and counseling prior to ATL. Hippocampal sclerosis (HS) is the most common pathological substrate in TLE and the best predictor of surgical success.^1^, ^4^ HS can be divided into three main subtypes based on pathological involvement (neuronal loss and gliosis) of specific hippocampal subfields (Type 1 HS—affecting subfields CA1 and CA4; Type 2 HS—predominantly affecting CA1 and Type 3 HS—predominantly affecting CA4).^5^, ^6^, ^7^ HS subtypes are shown to have unique postop seizure outcomes^5^, ^8^, ^9^, ^10^, ^11^ suggesting that localization of subfield pathology in vivo would improve patient selection for surgery.

MRI is currently one of the most important clinical tools for presurgical evaluation of TLE patients and is used to detect HS in vivo as measured by whole‐hippocampus reductions in volume and elevations of T2.^1^, ^12^, ^13^ Given the prognostic importance of assessing subfield‐specific abnormalities, many studies have now examined hippocampal subfield volumetry in TLE, for example refs.^14^, ^15^ Although these results show promise for the diagnosis of HS subtypes, many of these studies have relied on high field MRI (>3 T), limiting clinical applicability. Moreover, ex vivo surgical resection studies indicate that HS is also associated with re‐organization of tissue microstructure^16^, suggesting that complementary markers (ie, diffusion) may provide additional insight into microstructural changes associated with this pathological process.

Diffusion tensor imaging (DTI) indirectly evaluates the integrity of the neuronal microenvironment^17^, ^18^ and has been used extensively to study white matter connectivity in TLE—for review, see.^19^ DTI has also been applied to the hippocampus, showing elevated MD in the ipsilateral hippocampus of TLE patients with HS,^20^, ^21^, ^22^, ^23^, ^24^, ^25^, ^26^, ^27^, ^28^ and correlating with lower pyramidal cell density in CA4/DG on histology.^29^ However, all of these studies used low‐resolution acquisitions (6‐16 mm^3^ voxels) which limits analysis to the whole‐hippocampus or requires co‐registration with T1 and extensive postprocessing to examine regional changes within the hippocampus. Recent diffusion MRI protocol advances have enabled the acquisition of 1 mm isotropic DTI of the hippocampus in a clinically feasible scan time of under 6 minutes.^30^ The 6‐16× improvement in spatial resolution relative to previous studies demonstrates striking advantages in visualization of internal hippocampal architecture, as well as regional quantification of diffusion parameters within the hippocampus.

The purpose of this study was to use high‐resolution 1 mm isotropic DTI of the hippocampus in TLE patients and healthy controls in order to (a) determine if qualitative evaluation of internal architecture on mean DWI is sufficient to detect hippocampal sclerosis and (b) investigate regional diffusion abnormalities within the hippocampus that vary across TLE patients. In addition, whole‐hippocampus measures of diffusion, volume, and T2 will assess the interrelationships between these commonly acquired hippocampal parameters within subjects and provide global measures for comparison to past literature.

## 2 MATERIALS AND METHODS

### 2.1 Participants

This study enrolled 19 controls (ages 18‐70 years; mean 44 ± 14 years; 14 females) and 18 patients with TLE (18‐67 years; mean 42 ± 14 years; 11 females). Controls were recruited via word of mouth or advertising and were screened for self‐reported history of neurological disorders, seizure history, and contraindications to MRI. TLE patients were recruited from the University of Alberta Hospital Epilepsy Clinic via referral by their epileptologist. All participants provided written informed consent prior to participation, and the study was approved by the Health Research Ethics Board at the University of Alberta.

TLE patients were subdivided into unilateral HS (n = 8), bilateral HS (n = 3), and non‐HS TLE (n = 7) based on qualitative assessment of clinical MRIs. Of the seven non‐HS TLE subjects, one had a low‐grade tumor, one had a cavernous hemangioma, and the remaining subjects had no obvious structural lesion and no evidence of HS on clinical MRI. TLE subgroups did not differ by disease duration or age of onset, which ranged from 2‐53 and 0‐55 years, respectively. All patients were taking anticonvulsive medications. Four TLE patients underwent anterior temporal lobe resection after their research MRI scan, providing both imaging and histology in these subjects. One of these subjects had a tumor but no evidence of HS on MRI whereas the remaining three surgery subjects had evidence of unilateral HS on clinical MRI. See tables S1 and S2 for additional demographic and clinical details.

### 2.2 Cognitive testing

A subset of 13 TLE and 15 control participants were administered the NIH Toolbox Cognition battery (www.healthmeasures.net;^31^) including Picture Sequence Memory and Rey Auditory Verbal Learning on the same day as their MRI scan (remaining subjects were not administered cognitive testing due to time constraints). Group differences in raw scores were tested using ANCOVA, controlling for age. Hypothesis‐driven correlations between left whole‐hippocampus MD/volume and verbal memory (Rey Auditory Verbal Learning), and right whole‐hippocampus MD/volume and visual memory (Picture Sequence Memory) were tested in the TLE group, using partial correlations controlling for age. This hypothesis‐driven correlation was tested given past literatures suggesting right hippocampal involvement in visuospatial memory and left hippocampal involvement in verbal memory.^32^

### 2.3 Image acquisition & processing

Diffusion images were acquired on a 3T Siemens Prisma with single‐shot 2D EPI (GRAPPA R = 2; 6/8 PPF; A/P phase encode), FOV = 220 × 216 mm^2^, matrix = 220 × 216, 20 1 mm axial‐oblique slices aligned along the length of the hippocampus with no gap, 1 × 1 × 1 mm^3^ with no interpolation, TE = 72 ms, TR = 2800 ms, 10 averages of 10 gradient directions at *b* = 500 s/mm^2^ and 10 b0s acquired in 5:18 minutes as per Treit *et al*.^30^ Other scans included (a) 0.85 × 0.85 × 0.85 mm^3^ T1‐weighted MPRAGE (whole brain) in 3:39 minutes, (b) 0.5 × 0.5 × 1 mm^3^ axial T2‐weighted images with TE = 52 ms, TR = 5440 ms, 20 slices in 4:26 minutes, and (c) T2 relaxometry map with 16 echoes (TE = 10.7‐171.2 ms with 10 ms inter‐echo spacing), TR = 3560 ms, 1.1 × 1.1 × 1 mm^3^, 20 slices in 5:47 minutes. Both T2‐weighted and T2 relaxometry scans were acquired with alignment along the hippocampus identical to the diffusion acquisition.

Gibbs ringing, motion/distortion correction, and tensor calculation was performed in ExploreDTI v4.8.6. T2 maps were computed from multi‐echo spin echo data using extended phase graph (EPG)‐based indirect and stimulated echo compensation^33^ modified to use slice profiles approximated using the Shinnar‐Le Roux algorithm, as previously described.^34^

Hippocampi were manually segmented on mean DWIs in native space by a single user (author ST) in ITK‐snap v3.6.0 to yield volumes, FA and MD of bilateral hippocampi. An MD threshold of 2.0 × 10^−3^ mm^2^/s was used to exclude voxels containing primarily CSF for MD and FA measurements. Hippocampal ROIs were re‐drawn manually (author GL) on T2‐weighted images (with the same resolution and acquired in the same plane). A threshold of 200 ms was applied to the T2 maps in order to eliminate voxels that contain primarily CSF.

### 2.4 Qualitative image analysis

The mean *b* = 500 s/mm^2^ DWI of each subject in both groups was qualitatively evaluated by a single‐blinded rater (author DWG) to independently classify hippocampi as normal or sclerotic based on presence/absence of the stratum lacunosum moleculare (SLM). This qualitative evaluation was compared to clinically assigned HS classifications (or group in the case of controls) to calculate the sensitivity and specificity of HS identification with DWI. False negatives were defined as hippocampi with a clinical diagnosis of HS (from prior clinical MRI imaging) that were classified as “normal” on evaluation of the mean DWI. Likewise, false positives were cases that were classified as “HS” on evaluation of the mean DWI, with no clinical diagnosis of HS from prior clinical MRI imaging.

Intrarater reliability of this qualitative evaluation was determined by blinded reevaluation of a subset of 20 randomly selected subjects by the same rater (author DWG). Likewise, interrater reliability was determined by having a second rater (author ST) perform a blinded evaluation of 20 additional randomly selected subjects.

### 2.5 Quantitative image analysis

Analysis of covariance (ANCOVA) tested for omnibus effects of group (control, non‐HS TLE, unilateral HS and bilateral HS) on each continuous variable (whole‐hippocampus volumes, FA, MD and T2), controlling for age. Post hoc pairwise comparisons tested differences between each subgroup. To further visualize group differences, individual data were plotted against the control group mean and 2 SD boundaries. Relationships between whole‐hippocampus volume, MD, age of seizure onset and disease duration were tested using Pearson’s correlations.

### 2.6 Histology

Four of the TLE patients underwent ATL including resection of the anterior hippocampus (head and body) as part of their clinical care. As en bloc resection was not performed, it was not possible to precisely determine the localization of histological slides in relation to the imaging data. Based on the standardized surgical approach used by the treating neurosurgeon, the histological specimen analyzed was consistently obtained from the posterior head and anterior body of the hippocampus. Specimens underwent histological staining and were assessed by a pathologist as part of routine clinical care. Stains included NeuN as a marker of neuronal loss, glial fibrillary acidic protein (GFAP) for reactive gliosis, and luxol fast blue (LFB) for myelin density. Presence and degree of neuronal loss and gliosis in each of CA1 and CA4 and an assignment of HS subtype was given by the reviewing pathologist.

## 3 RESULTS

### 3.1 Qualitative evaluation of mean DWI

**Figure 1:**
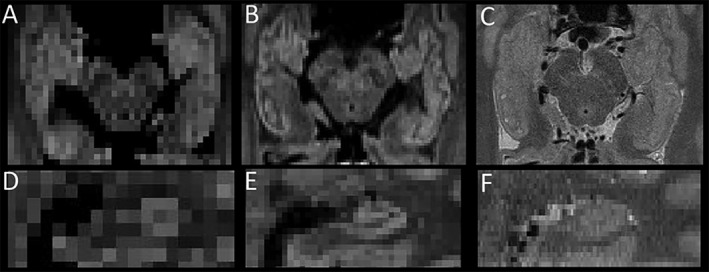
Mean diffusion‐weighted images (DWI; *b* = 500 s/mm^2^) from a standard 2 mm isotropic DTI (A—axial, D—coronal) and 1 mm isotropic DTI acquisition (B—axial, E—coronal) are shown relative to a 0.5 × 0.5 × 1 mm^3^ T2‐weighted image (C—axial, F—coronal) in the same 33‐year‐old female control from Treit et al 2018 Neuroimage. 1 mm isotropic DTI acquisition provides better visualization of gross structure (digitations, shape, etc.), internal architecture (eg, stratum lacunosum moleculare; SLM) and differentiation from surrounding structures (eg, CSF, amygdala) than the other two scans.

**Figure 2:**
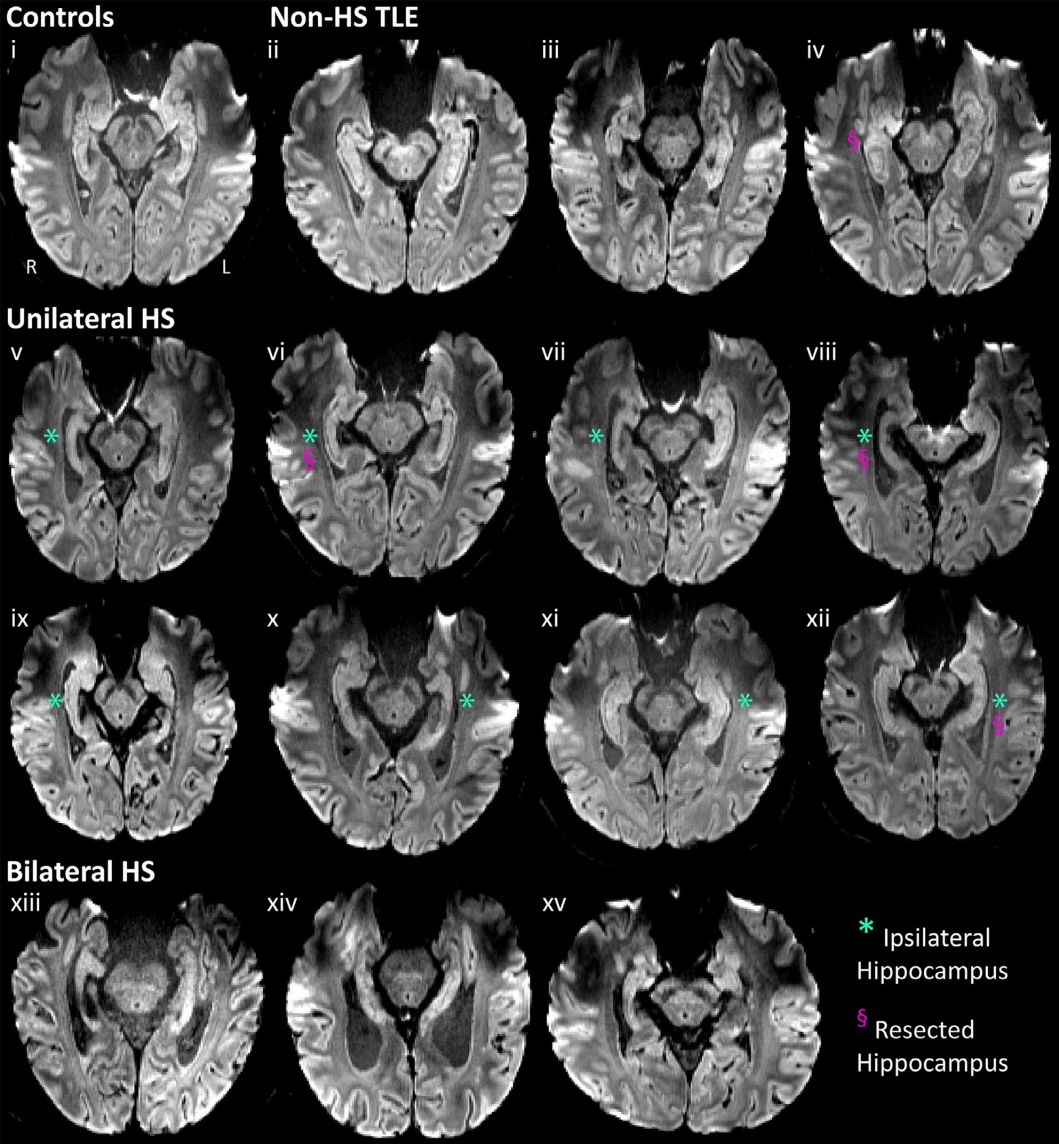
Mean diffusion‐weighted images (DWI), for one control subject (i), three non‐HS TLE patients (ii, iii, iv) and all eight unilateral (v‐xii) and three bilateral HS patients (xiii‐xv). Substantial variability in hippocampal atrophy and presence/absence of substructure is seen between subjects. In particular, the SLM is visible in control and nonlesional examples, but is notably absent on the ipsilateral side of most unilateral HS patients (eg, v, vii, viii) and bilaterally in patients xiii, xiv, and xv. Note groupings are made based on clinical classification of HS. The ipsilesional hippocampus of unilateral HS patients is marked with *; likewise the hippocampus that was subsequently resected in the four surgical patients is marked with §.

Mean DWI provided excellent visualization of gross hippocampal morphology (eg, size and shape, digitations), delineation from surrounding structures including the amygdala, and improved contrast of internal hippocampal architecture (eg, SLM) as compared to lower resolution DTI and T2 (Figure 1). Marked reductions of SLM contrast were observed within sclerotic hippocampi of TLE patients, seen as loss of the dark band on mean DWI (eg, Figure 2 v,xii). Blinded visual evaluation of mean DWIs resulted in correct classification of 12/14 hippocampi with HS (6/8 ipsilesional hippocampi of unilateral HS patients and 6/6 hippocampi of bilateral HS patients), yielding 86% sensitivity. Likewise, 56/60 hippocampi without HS were correctly classified (36/38 control hippocampi, 13/14 non‐HS hippocampi, and 7/8 contralesional hippocampi of unilateral TLE subjects), yielding 93% specificity. In repeated qualitative analyses, 37/40 hippocampi were consistently classified between evaluations within rater (author DWG) and 39/40 hippocampi were consistently classified between raters (authors DWG and ST) indicating excellent intra‐ and interrater reliability of 93% and 98%, respectively.

### 3.2 Whole‐hippocampus volume, FA, MD and T2

**Figure 3:**
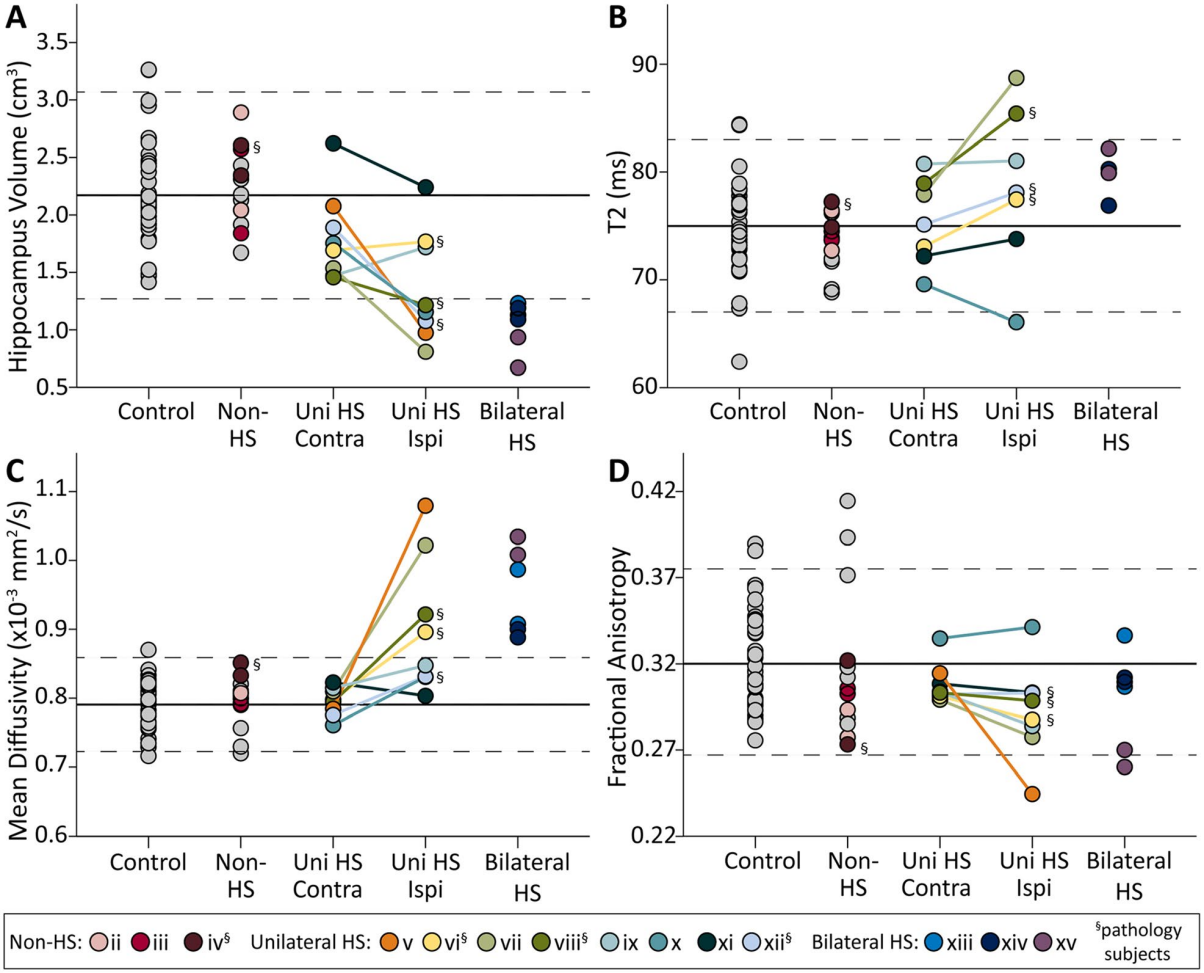
Volume (A), T2 (B), MD (C), and FA (D) for bilateral hippocampi in all 37 subjects, plotted against control mean (solid line) and ±2 SD (dotted lines). Note that only TLE subjects found in Figures 2, 4, 5, and 6 are colored here to enable cross‐referencing between figures. Bilateral hippocampi of unilateral HS patients are shown separately (linked with lines) given expected differences in pathology between ipsilesional and contralesional hemispheres. As expected, non‐HS TLE subjects and the contralesional hippocampus of unilateral HS patients typically fell within 2 SD of the control mean for all measures. Conversely, ipsilesional hippocampi and both hippocampi of bilateral HS patients had much lower volumes and higher MD than controls (>2 SD) in many individuals, but these deviations were less prevalent for T2 and FA in the TLE subjects. The four subjects with ipsilateral hippocampus tissue histology post‐surgery are indicated by §.

ANCOVA revealed significant effects of group for all MRI measures: whole‐hippocampus volume (*F *= 15.0, *P *< 0.001), FA (*F *= 3.0, *P *= 0.023), MD (*F *= 24.1, *P *< 0.001), and T2 (*F *= 2.8, *P *= 0.032; Figure 3, Table S1). Specifically, volumes of sclerotic hippocampi were ~36‐55% lower than contralateral, non‐HS, or control hippocampi. Interestingly, contralateral hippocampi of unilateral HS patients were also ~18% smaller than the control and non‐HS groups (which were not different from each other). MD was highest in bilateral HS hippocampi (~20% higher than controls), followed ipsilateral hippocampi of unilateral HS patients (~14% higher than controls), while contralateral hippocampi, non‐MTS, and control groups were not different from each other. FA was 6‐9% lower and T2 was 5‐7% higher in sclerotic hippocampi relative to contralateral hippocampi of unilateral patients, non‐HS and controls, which were not significantly different from each other for either measure. There were no significant correlations between volume or MD of sclerotic hippocampi with disease duration or age of seizure onset.

When evaluated individually, the volume and MD of all 22 nonsclerotic hippocampi (14 non‐HS TLE patients + contralateral hippocampi of 8 unilateral HS patients) were within 2 SD of controls (Figure 3). Of the hippocampi with HS (6 bilateral + 8 unilateral = 14 total), both volume and MD were >2 SD from controls in 9/14 sclerotic hippocampi, volume (but not MD) was >2 SD from controls in 2/14 sclerotic hippocampi, MD (but not volume) was >2 SD from controls in 1/14 sclerotic hippocampi. The remaining 2 sclerotic hippocampi were within 2 SD for both volume and MD. A post hoc correlation between MD and volume revealed no correlation in nonsclerotic hippocampi, but a negative correlation in sclerotic hippocampi (Figure S1). Of note, significant scatter remains even within the significant negative correlation, suggesting a lack of correspondence for several subjects. FA was >2 SD below controls for only 2/14 hippocampi, and T2 was >2 SD higher than controls in only 3/14 sclerotic hippocampi.

### 3.3 Whole‐hippocampus MD and volume versus memory

ANCOVA revealed significant group differences for both Rey Auditory Verbal Learning (*F *= 15.6, *P *= 0.001) and Picture Sequence Memory (*F *= 32.3, *P *< 0.001; Table S1). Partial correlations (controlling for age) revealed a significant negative correlation between verbal memory and left hippocampal MD (*R *= −0.745, *P *= 0.005; Figure S2) but not volume (*R *= 0.086, *P *= 0.760) in the TLE group. Correlations between volume and verbal memory were repeated including intracranial volume as a covariate, but remained nonsignificant. There were no significant correlations between right hippocampus MD or volume and visual memory.

### 3.4 Heterogeneous regional MD abnormalities within the hippocampus

**Figure 4:**
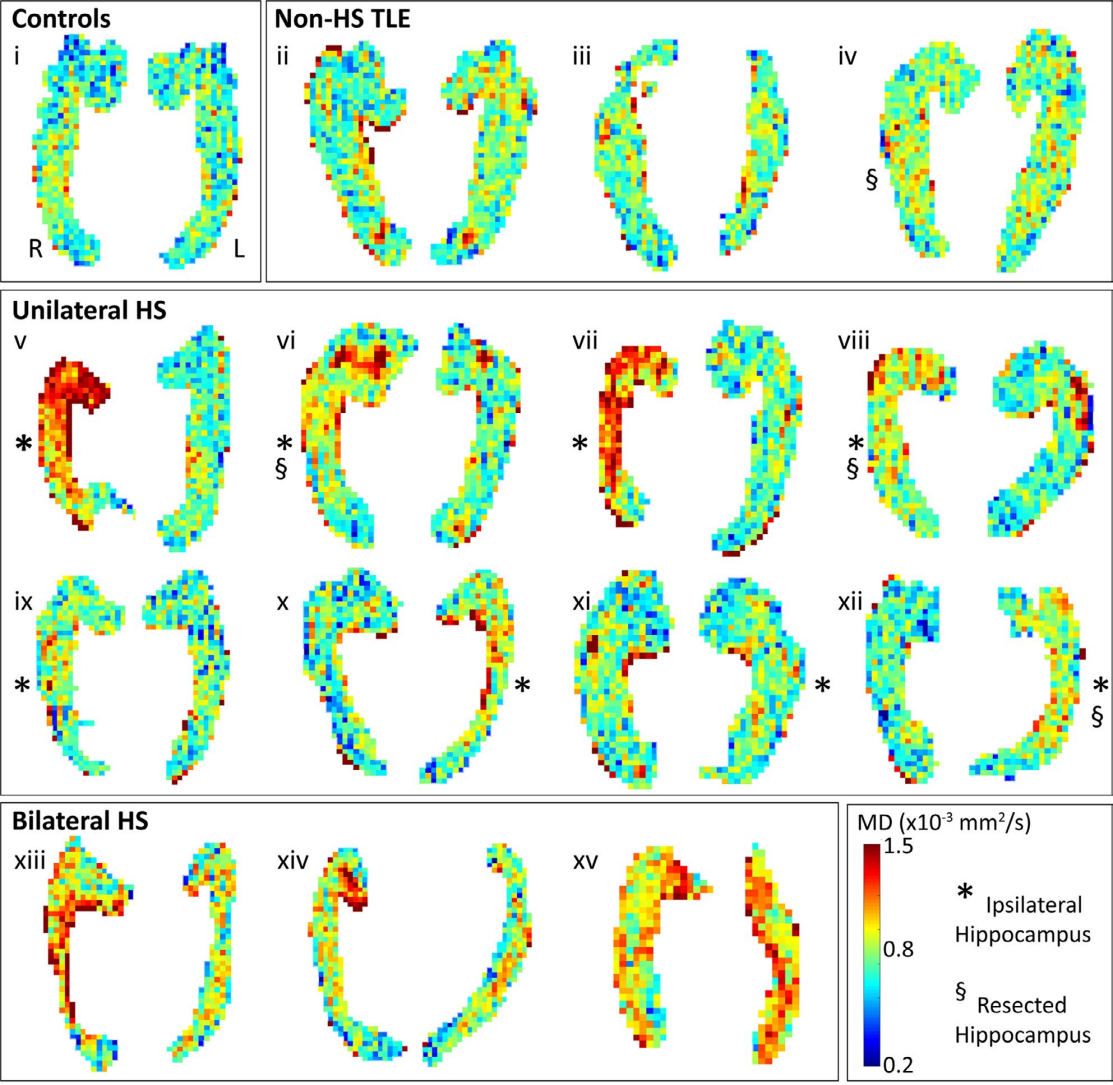
MD maps of both hippocampi from representative control (i), non‐HS TLE patients (ii‐iv), unilateral HS (v‐xii), and bilateral HS patients (xiii‐xv), as in Figure 2 which showed the mean DWI in the same subjects. The MD maps in non‐HS TLE are comparable to control (mostly green ~0.8 × 10^−3^ mm^2^/s), whereas MD is abnormally high in a heterogenous pattern across sclerotic hippocampi in unilateral and bilateral HS TLE patients. For example, widespread elevations are seen throughout the hippocampus in the ipsilesional hippocampi (indicated by *) of v and vii, as well as bilaterally in xv, whereas more regional elevations are noted in the hippocampal head in vi (ipsi) and xiv (right). Note that MD maps are not scaled to size.

The 1 mm resolution of the DTI protocol enabled visualization of considerable heterogeneity in diffusion abnormalities within the hippocampus of TLE patients (Figure 4). The MD maps in non‐HS TLE were comparable to controls, while regional elevations of MD (>4 SD of control values) are visually evident in all patients with either unilateral or bilateral HS, including in hippocampi with normal volume relative to controls (eg, Figure 4 patient vi). Within the unilateral HS group, some patients showed widespread MD elevations throughout the entire head body and tail of the ipsilesional hippocampus (eg, Figure 4 v, vii), whereas others showed more focal elevations such as in the medial head (Figure 4 vi) or lateral body (Figure 4 viii) of the ipsilesional hippocampus. Of note, focal regions of elevated MD were observed in the contralateral hippocampus of several patients (Figure 4 vi, viii), which may indicate bilateral (albeit asymmetric) disease. Bilateral HS patients had increased MD throughout both hippocampi, albeit with asymmetry in some patients (eg, right body in Figure 4 xiii, right head in Figure 4 xiv). Interestingly, the bilateral HS patient with the most obvious right‐sided asymmetry (Figure 4 xiii) also had right‐lateralized telemetry (see Table S2). MD abnormalities were most prominent anteriorly with MD preserved in the hippocampal tail for most, with the exception of the bilateral HS TLE group.

### 3.5 Histology comparisons to presurgical MD maps

**Figure 5:**
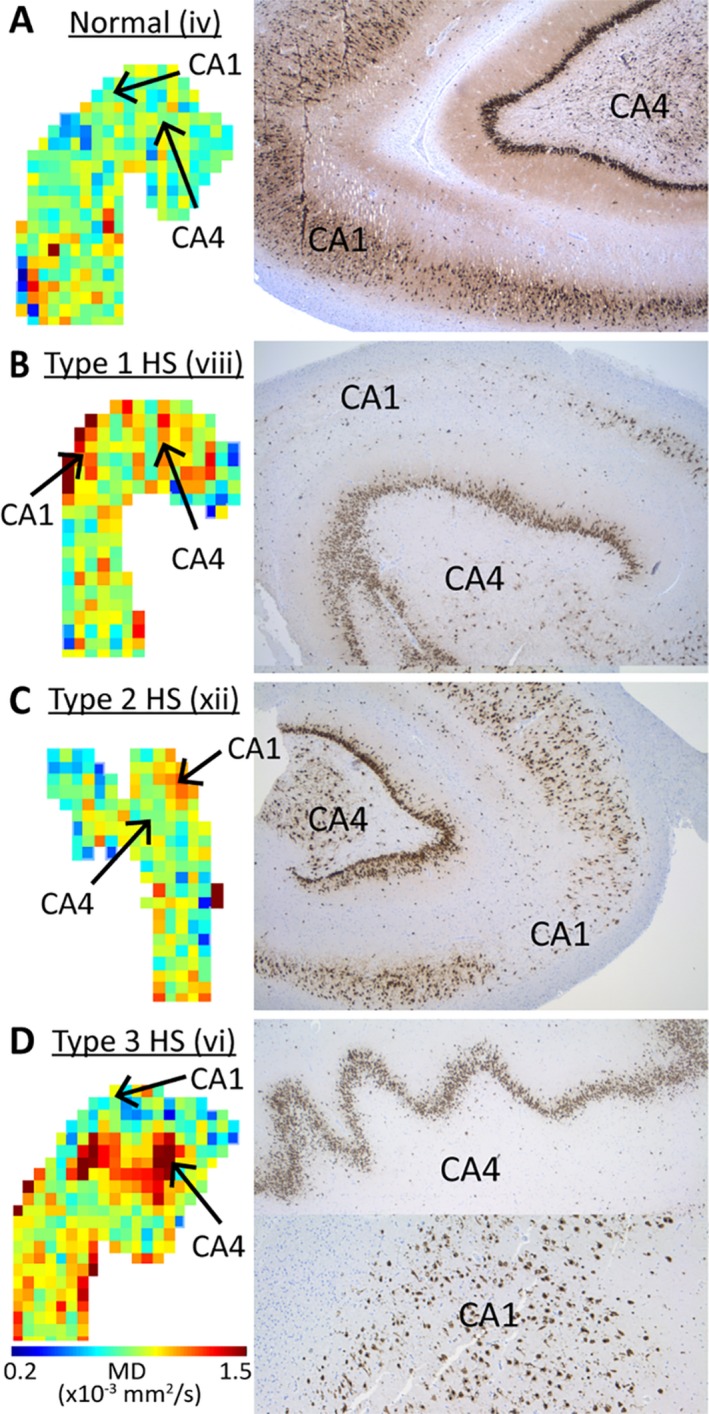
Presurgical MD maps (axial) and NeuN histology (coronal) of four TLE patients who underwent surgical resection of the hippocampus: A, nonlesional hippocampus showing typical MD values (~0.8 × 10^−3^ mm^2^/s; mostly green) and neuronal density (dark brown) throughout the hippocampus; B, type 1 hippocampal sclerosis (HS) where elevated MD in both CA1 and CA4 fit with neuronal loss in both CA1 and CA4, (C) type 2 HS with elevated MD in only CA1 matching marked neuronal loss in CA1 but not CA4, and (D) type 3 HS with elevated MD in only CA4 matching neuronal loss in CA4 but not CA1.

Surgical histology was available in four subjects (one with a brain tumor and three with evidence of unilateral HS on clinical MRI) with the specimen provided by the neurosurgeon consisting of a portion of the hippocampus in the region of the posterior head and anterior body. The patient with a ganglioma (iv) had normal hippocampal subfields with no evidence of HS. Of the three patients with evidence of unilateral HS on MRI, patient viii had dramatic cell loss in CA1 and CA4 (Type 1 HS), patient xii had dramatic neuronal loss of CA1 with sparing of CA4 (Type 2 HS), and patient vi had dramatic cell loss in CA4 but sparing of CA1 (Type 3 HS; Figure 5).

**Figure 6:**
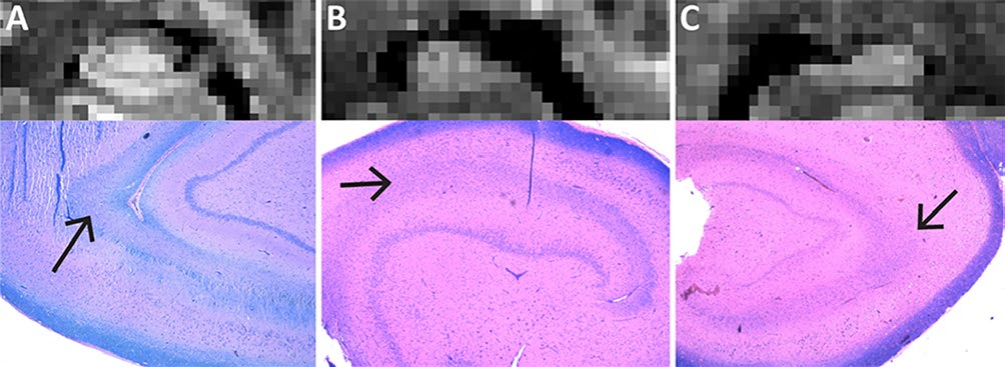
Coronal mean DWI and corresponding histological sections, stained with luxol fast blue (LFB) of three of the four patients from Figure 5. A, The nonlesional TLE subject (patient iv) shows a visible SLM on mean DWI and normal myelin staining with LFB, whereas the two subjects with type 1 HS (B, patient viii) and 2 HS (C, patient xii) do not have a clearly defined SLM on mean DWI and demonstrate reduced myelin staining on LFB. Note that tissues were stained in different batches and therefore degree of blue versus pink varies from specimen to specimen. LFB staining is not shown for the patient with type 3 HS as the tissue sample provided for LFB staining was of poor quality and it was therefore not possible to confidently assess the degree of myelin staining in the SLM.

Histological samples demonstrate excellent spatial correspondence between subfield pathology of neuronal loss on NeuN and gliosis on GFAP (data not shown) with regional MD abnormalities (Figure 5). As expected, patient iv who had TLE secondary to a brain tumor was found to have a normal MD map and no underlying hippocampal pathology (Figure 5A). Conversely, all three remaining hippocampi were found to have subfield pathology consistent with the regional abnormalities observed on MD maps. Specifically, subject viii had elevated MD throughout the head and upper body with histology typical of type 1 HS including neuronal loss and gliosis in CA1 and 4 (Figure 5B), subject xii had a focal region of elevated MD in the lateral head with histology typical of type 2 HS including neuronal loss and gliosis in CA1 but not CA4 (Figure 5C), and subject vi had a focal region of elevated MD in the mesial head with histology typical of type 3 HS including neuronal loss and gliosis in CA4 but not CA1 (Figure 5D). LFB staining revealed normal myelin density in the SLM of the non‐HS subject, who also had a visible SLM on mean DWI (Figure 6A) and loss of myelin in the subjects with type 1 and 2 HS who likewise had no visible SLM on mean DWI (Figure 6B,C).

## 4 DISCUSSION

This first application of high‐resolution (1 mm isotropic) DTI of the hippocampus in TLE (acquired in a clinically feasible scan time of ~5 minutes at 3T) enabled both qualitative and quantitative analysis of diffusion parameters within individuals. The 6‐16× greater spatial resolution compared to past DTI literature in TLE allowed for qualitative evaluation of internal architecture that is not visible on DWI at lower resolutions. Specifically, loss of a visible SLM on DWI was demonstrated to identify patients with HS with excellent sensitivity, specificity, and reliability. Moreover, preliminary histology evidence suggested that loss of SLM on DWI is accompanied by reduced myelin staining, in keeping with ex vivo diffusion MRI work suggesting that HS is associated with abnormal fiber bundle organization in the SLM.^16^ Loss of internal architecture on anatomical imaging (usually T2) is a hallmark feature of HS and can be found in the absence of atrophy and increased T2 signal.^35^, ^36^ This work suggests that high‐resolution DWI may provide complementary contrast for evaluating loss of internal architecture as a marker of HS.

Histological studies in TLE have demonstrated regional variability of hippocampal pathology within different subfields,^5^ along the length of the hippocampus^37^ and involving the contralateral hippocampus^38^. Ex vivo diffusion MRI studies of healthy hippocampal tissue suggest that FA and MD vary regionally with excellent correspondence with cellular histology,^39^ providing a basis for evaluating diffusion parameter changes associated with cellular pathology in vivo. Going beyond whole‐hippocampus diffusion measures, this study uncovered striking regional heterogeneity of MD within the hippocampus of subjects with HS (with focal MD “hotspots” >4 SD of control values) in keeping with histological literature finding regional variation in neuronal loss in HS.^37^ Variability was observed between subjects, with some showing widespread areas of high MD (subjects v and vii) while others demonstrate more focal abnormalities restricted to anterior mesial (subjects vi and xiv), posterior (subject xiii), or lateral (subjects viii and xii) regions of the ipsilesional hippocampus (Figure 4). Histological correlations were possible in four subjects. As histology was restricted to a limited specimen (rather than en bloc) from the anterior ipsilateral hippocampus, it was not possible to perform a precise direct correlation between regional MD changes and histology. However, the observed spatial correspondence between regional hippocampal MD elevation and histological markers (neuronal cell density and gliosis) suggest that high‐resolution DTI has the potential to detect hippocampal subfield pathological changes (Figure 5), which given the observed correlations between HS subtype and surgical outcomes in histology studies^5^ could improve the preoperative prediction of surgical outcomes.

In addition to ipsilateral pathology, regional MD abnormalities in the contralateral hippocampus were also observed in the absence of changes to contralateral whole‐hippocampus MD (subjects vi and viii; Figure 3) and thus would not be detected with lower resolution methods. Variability of HS pathology in the contralateral hippocampus^38^ and postsurgical contralateral hippocampus volume and diffusion changes observed in vivo^40^ suggest that abnormalities in the unresected hippocampus may impact seizure and memory outcomes and should be investigated preoperatively using methods such as diffusion imaging.

Our demonstration of increased whole‐hippocampus MD in TLE with HS is consistent with previous low‐resolution literature, for example,^21^, ^22^, ^24^, ^25^, ^27^ and suggests that elevated hippocampal MD is a robust marker of the structural changes, providing complementary information to volume about underlying pathology associated with HS. Longitudinal imaging is needed to elucidate the relative time‐course of MD and volume changes associated with HS, as well as the trajectory of their association with memory, seizure frequency, and surgical outcomes. Left hippocampus MD (but not volume) correlated with verbal memory in the TLE group, indicating that changes in diffusivity may be more tightly linked to cognition than volume. Although several previous studies have found correlations between hippocampal volume and memory in TLE, for example,^32^, ^41^ few have examined relationships between hippocampal diffusion and memory^26^ and none have looked at both measures in the same sample. An understanding of the pathological substrate of memory impairment in TLE is critical in order to identify patients at risk for worsening memory impairments following anterior temporal lobectomy.

This study demonstrates the potential utility of 1 mm isotropic hippocampal DTI (acquired in a clinically feasible scan time at 3T) for the diagnosis of HS and the characterization of HS subtype in TLE in vivo. Importantly, both qualitative evaluation of internal architecture and regional changes in diffusion properties within the hippocampus can be evaluated in native space with minimal processing, which could provide a clinically feasible tool for improved prediction of surgical outcomes in medically intractable patients.

## CONFLICT OF INTEREST

None of the authors have any conflict of interest to disclose. We confirm that we have read the Journal’s position on issues involved in ethical publication and affirm that this report is consistent with those guidelines.
