# Supplementary figures and images for: Regional hippocampal diffusion abnormalities associated with subfield‐specific pathology in temporal lobe epilepsy
Source: Epilepsia Open. 2019 Sep 13;4(4):544–54. doi: 10.1002/epi4.12357 (PMC6885671; doi:10.1002/epi4.12357)

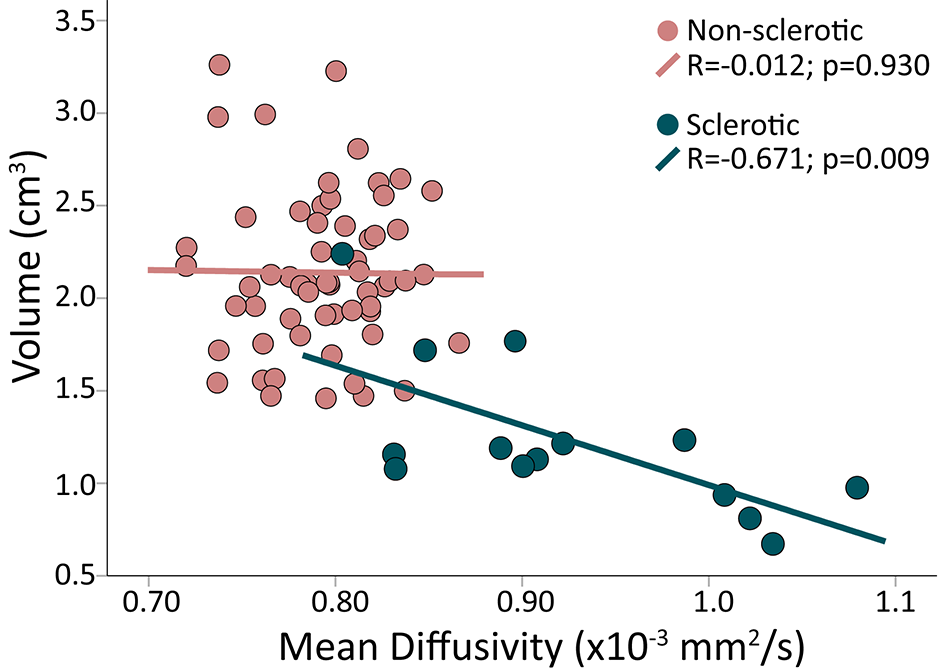

Supplement: Supplementary file 1 [file EPI4-4-0-s001.tif]

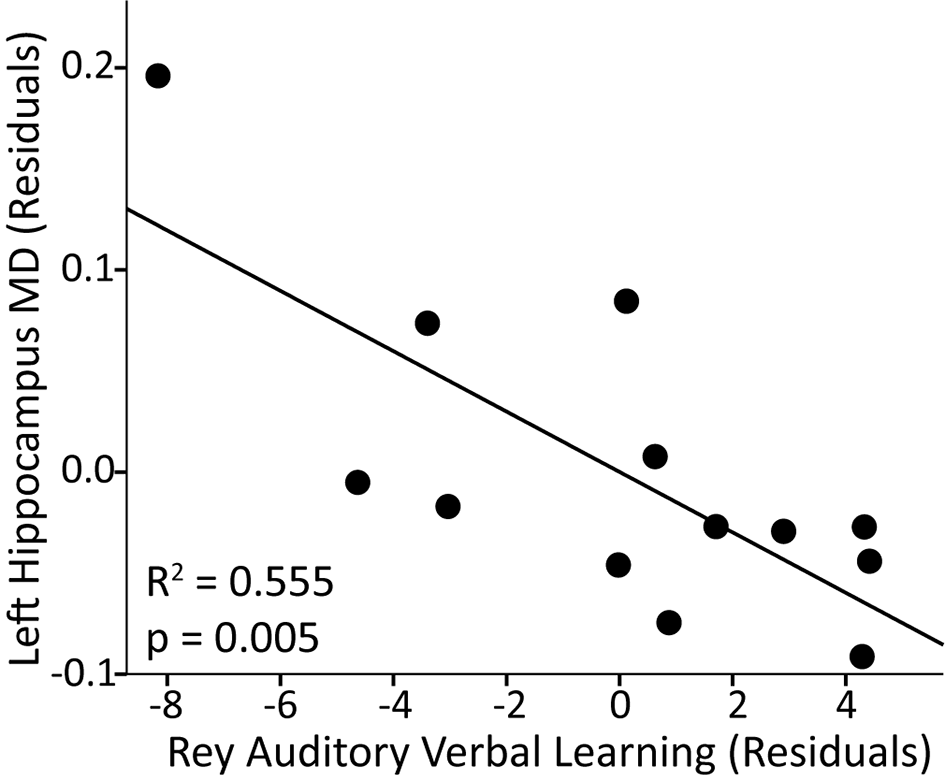

Supplement: Supplementary file 2 [file EPI4-4-0-s002.tif]
