# Supplementary material for: Regional hippocampal diffusion abnormalities associated with subfield‐specific pathology in temporal lobe epilepsy
Source: Epilepsia Open. 2019 Sep 13;4(4):544–54. doi: 10.1002/epi4.12357 (PMC6885671; doi:10.1002/epi4.12357)
Supplement: Supplementary file 3 [file EPI4-4-0-s003.docx]

**SUPPORTING INFORMATION:**

**Supporting Information Table 1:** Whole Hippocampus MRI Measures, Clinical and Cognitive Scores by Group

|  |  |  | Temporal Lobe Epilepsy | |  |
| --- | --- | --- | --- | --- | --- |
|  | Control (n=19) | Non-HS (n=7) | Unilateral HS  (n=8) | | Bilateral HS (n=3) |
| *Whole Hippocampus MRI Measures^a^* | | | | | |
|  |  |  | *Contra* | *Ipsi* |  |
| Volume (cm^3^)^b^ | 2.2 ± 0.5 | 2.2 ± 0.3 | 1.8 ± 0.4^*^ | 1.4 ± 0.5^*^ | 1.0 ± 0.2^*^ |
| FA | 0.32 ± 0.03 | 0.32 ± 0.04 | 0.31 ± 0.01 | 0.29 ± 0.03^*^ | 0.30 ± 0.03^*^ |
| MD (x10^-3^ mm^2^/s) | 0.79 ± 0.03 | 0.80 ± 0.04 | 0.80 ± 0.02 | 0.90 ± 0.10^*^ | 0.95 ± 0.06^*^ |
| T2 (ms) | 75 ± 4 | 74 ± 3 | 75 ± 4 | 79 ± 7^*^ | 80 ± 2^*^ |
| *Clinical & Cognitive Measures* | | | | | |
| Age (years): Median (range) | 46 (18-67) | 46 (18-54) | 47 (20-70) | | 49 (36-58) |
| Sex (M/F) | 5/14 | 3/4 | 3/5 | | 1/2 |
| Disease Duration (years) | n/a | 7 (2-28) | 14.5 (3-53) | | 18 (9-53) |
| Age of Onset (years) | n/a | 31 (14-47) | 22 (0-55) | | 27 (5-35) |
| Picture Sequence Memory (Raw) | 19 ± 6 | 10 ± 4^*^ | 9 ± 4^*^ | | 2 ± 1^*^ |
| Rey Auditory Verbal Learning (Raw) | 27 ± 4 | 23 ± 2^*^ | 21 ± 5^*^ | | 17 ± 3^*^ |

^a^ Left and right values averaged for all subjects except unilateral HS patients, where ipsilateral and contralateral hippocampi are presented seperately

^b^Volumes measured manually on mean DWI are lower than typically observed from segmentation of T1-weighted images, presumably due to exclusion of subiculum on mean DWI analysis.

*Significant difference (p<0.05) from controls based on pairwise comparisons from ANOVA for clinical measures and ANCOVA controlling for age for MRI and cognitive measures.

**Supporting Information Table 2:** Clinical and Demographic data for TLE patients

| **Subject** | **Age (years)** | **Sex** | **Age at Onset (years)** | **Epilepsy Duration (years)** | **HS Classification**  **(clinical MRI)** | **Telemetry**  **Lateralization** |
| --- | --- | --- | --- | --- | --- | --- |
| ii | 34 | Male | 31 | 3 | Non HS | Left |
| iii | 54 | Female | 47 | 7 | Non HS | Left |
| iv | 18 | Female | 16 | 2 | Non HS | None |
| v | 47 | Female | 9 | 38 | Unilateral – Right | Right |
| vi | 20 | Male | 17 | 3 | Unilateral – Right | Right |
| vii | 70 | Female | 17 | 53 | Unilateral – Right | Right |
| viii | 60 | Female | 55 | 5 | Unilateral – Right | Right |
| ix | 27 | Female | 0 | 27 | Unilateral – Right | Right |
| x | 43 | Male | 34 | 9 | Unilateral – Left | Left |
| xi | 49 | Male | 44 | 5 | Unilateral – Left | Left |
| xii | 47 | Female | 27 | 20 | Unilateral – Left | Left |
| xiii | 58 | Male | 5 | 53 | Bilateral | Right |
| xiv | 53 | Female | 35 | 18 | Bilateral | Bilateral |
| xv | 36 | Female | 27 | 9 | Bilateral | None |
| xvi* | 32 | Female | 14 | 18 | Non HS | Bilateral |
| xvii* | 46 | Male | 18 | 28 | Non HS | Bilateral |
| xviii* | 50 | Male | 44 | 6 | Non HS | Right |
| xix* | 50 | Female | 32 | 18 | Non HS | Left |

*Subject data not identified in figures
